# Dyspepsia Treated with Raw Meat

**Published:** 1871-05

**Authors:** 


Dyspepsia treated with Raw Meat.—So common is dyspepsia that, according to Chomel, a fifth of all his patients were affected with it. Nor is this extraordinary when it is remembered that when the digestive system loses in some degree its vigor and activity, the other functions are irregularly and languidly performed; the reparative materials soon become defective in quantity and quality; the blood becomes poor, loses its plasticity, and circulates through the several tissues without nourishing them; the constitution begins to be profoundly disturbed; and soon upon the primary dyspepsia are grafted gastralgia, anæmia, chlorosis, nervous affections—in a word, every affection that arises from and again reacts in producing a defect of vital resistance. The danger is great unless the treatment be appropriate. What has been held to be appropriate has varied from time to time: new bitters, then tonics, and now again ferruginous compounds. Sometimes alkalies have been regarded as the universal panacea; and mere recently pepsine and strychnia have been vaunted as specifics. Still it would appear the really appropriate treatment has to be sought: and our newly established contemporary, the *Echo Belge*, from which we have extracted these remarks, reminds us of the old adage, "*telle nourriture, tel sang*," and adds that such as is the blood so is the force of resistance to the influence of disease. In all such cases it is of the highest importance to supply the economy in the easiest manner with the largest amount of alimentary materials. With this object in view, uncooked meat is especially indicated, and appears to have been somewhat largely prescribed by the French physicians, though the records of its success are comparatively few. To administer it properly it is necessary to attend to several points. The fillet should be preferred, as being the most delicate and the richest in muscular fibrin. It should be freed with the utmost care from fat and tendon. It should be finely minced, and then brayed in a mortar of wood or stone. When reduced to paste, it should be covered with sugar, gluten, or vegetable gelatine to overcome the repugnance with which it is at first naturally regarded. Some prefer to squeeze out the juice and swallow it mixed with a little rum or orange-flower water; whilst others again make it into boluses, and take it in slightly warmed beef tea or soup.

